# Masculinity, social context and HIV testing: an ethnographic study of men in Busia district, rural eastern Uganda

**DOI:** 10.1186/1471-2458-14-33

**Published:** 2014-01-13

**Authors:** Godfrey E Siu, Daniel Wight, Janet A Seeley

**Affiliations:** 1 MRC/UVRI Uganda Research Unit on AIDS, Entebbe, P.O. Box, 49, Entebbe, Uganda; 2 Child Health and Development Centre, College of Health Sciences Makerere University, P.O. Box, 6717, Kampala, Uganda; 3 School of International Development, University of East Anglia, NR4 7TJ Norwich, UK; 4 MRC Social & Public Health Sciences Unit, 4 Lilybank Gardens, G12 8RZ Glasgow, UK; 5 London School of Hygiene and Tropical Medicine, Keppel Street, WC1E7HT London, UK

## Abstract

**Background:**

Uptake of HIV testing by men remains low in high prevalence settings in many parts of Africa. By focusing on masculinity, this study explores the social context and relations that shape men’s access to HIV testing in Mam-Kiror, Busia district, rural eastern Uganda.

**Methods:**

From 2009–2010 in-depth interviews were undertaken with 26 men: nine being treated for HIV, eight who had tested but dropped out of treatment, six not tested but who suspected HIV infection and three with other health problems unrelated to HIV. These data were complemented by participant observation. Thematic analysis was undertaken.

**Results:**

There were two main categories of masculinity in Mam-Kiror, one based on ‘reputation’ and the other on ‘respectability’, although some of their ideals overlapped. The different forms of masculine esteem led to different motives for HIV testing. Men positioned HIV testing as a social process understood within the social context and relationships men engaged in rather than an entirely self-determined enterprise. Wives’ inferior power meant that they had less influence on men’s testing compared to friends and work colleagues who discussed frankly HIV risk and testing. Couple testing exposed men’s extra-marital relationships, threatening masculine esteem. The fear to undermine opportunities for sex in the context of competition for partners was a barrier to testing by men. The construction of men as resilient meant that they delayed to admit to problems and seek testing. However, the respectable masculine ideal to fulfil responsibilities and obligations to family was a strong motivator to seeking an HIV test and treatment by men.

**Conclusion:**

The two main forms of masculine ideals prevailing in Mam-Kiror in Busia led men to have different motives for HIV testing. Reputational masculinity was largely inconsistent with the requirements of couple testing, community outreach testing and the organisation of testing services, discouraging men from testing. Conversely, concern to perform one’s family roles as a respectable man meant accessing treatment to extend one’s life, which encouraged men to test. HIV support agencies should reflect on how various testing options might marginalise men from seeking testing services and address the barriers that hinder access.

## Background

HIV testing remains low in most parts of Sub-Saharan Africa (SSA), although there are wide variations between countries [1]. While there has been a remarkable increase in the overall prevalence of HIV testing in Uganda in recent years, the gender gap in testing seems to be increasing. Recent estimates from the Uganda AIDS Indicator Survey show that 66% of women and 45% of men have ever been tested [2]. The gender variation in national testing rates in Uganda appears to be consistent with trends observed in other eastern and southern African countries, such as Kenya, Tanzania and South Africa, where women are also more likely to test than men [3-7], as well as in West Africa [8].

A range of barriers to HIV testing has been identified in the literature, although often without consideration of gender. These include lack of confidentiality, stigmatizing beliefs and fear of discrimination in the event of a positive test, transport difficulties and low perceived risk of HIV infection [9-14]. There are, however, some exceptions to these, notably [7,15,16], who did a gender analysis of differences in testing in other parts of SSA and [17,18], who specifically focused on men’s testing in Uganda. The common finding reported by these studies is that there were some gender specific explanations for men’s lower rates of testing compared to women. Although it is often assumed that men tend to have more and riskier sexual relationships compared to women, men tend to underestimate their risk of HIV infection compared to women [19]. If men test due to their perceived risky sexual behaviour, the testers are most likely to be those with a history of paying for sex rather than other categories of men [18]. Fear of an HIV positive test and worries about disclosure and blame for bringing HIV into the family also discourage men from testing [17,19]. More testing programmes targeting women than men might also explain the gender difference. In many SSA countries there has been a national scale-up of women’s testing programmes, reducing access barriers for them [6,7]), yet generally doing little to accommodate men’s special testing needs [20].

While the literature on the role of gender is growing, most studies in this area have been quantitative and shed little light on the lived experiences of masculinity and how it influences testing. Although studies elsewhere have suggested that norms of masculinity, the societal expectation of what it means to a man [21], have an influence on HIV testing among men and have called for further research in this area [15], such research remains sparse in Uganda. Furthermore, existing qualitative research has tended to focus on the negative influences of masculinity and less on its positive role on HIV testing among men. The present article explores how various factors interact with themes of masculinity and either discourage or encourage men’s HIV test-seeking behaviour. This article draws from a larger study that examined the relationship between men’s uptake of HIV treatment and their masculinity in Mam-Kiror village in Busia district in rural eastern Uganda between 2009 and 2010. Mam-Kiror’ is a pseudonym for the study area.

### Theoretical framework

Empirical research on the relationship between masculinity and men’s health suggests that the health behaviour and practices that men adopt are often a means of constructing their gender [22]. The relational theories of gender argue that as social practices masculinity and femininity are produced and reproduced through people’s interactions and shaped by relations of power [23]. However, since it is constructed in multiple contexts and circumstances, masculinity is not a static concept; it is diverse and multiple and varies between and even within societies [24]. The recognition of the multiplicity and relational nature of masculinity is particularly relevant in this article as it draws attention to the possibility of diversity in health related practices and behaviour.

In our previous analysis we discussed in detail the prevailing constructs of masculinity in Mam-Kiror village [25]. We argued that there were essentially two forms of masculinity in Mam-Kiror, one based on the ideas of ‘reputation’ and the other on ideals of ‘respectability’ [26], with some ideals shared by both. Respectability was endorsed by the wider society, primarily women, some men, religious ministers and relatives of one’s wife (in-laws). It consisted of ideals such as marriage, fathering children and providing for them, sexual fidelity, respect for self and others and hard work. The reputational ideals were predominantly endorsed by men amongst themselves and included sexual prowess, fathering many children, physical strength, a work ethic, socialising with others and compulsory spending on leisure. For example, subscribing to the masculinity of (hetero) sexual achievement meant that men competed intensively for and often shared, sexual partners regardless of marital status and the importance of being the first man to establish a sexual relationship with a newcomer related to the reputation attained from others, who admired their seduction skill and/or use of money. However, the distinction between respectable and reputational masculinities was at times more complex. For instance, some men sometimes attempted to establish their respectability with other men, such as their affines. This suggests that in constructing their identities, men often drew from conflicting notions of masculinity and adopted the ideals that were appropriate to the social context, life stage and company they maintained. In this paper we examine how these dimensions of masculinity may influence HIV testing behaviour among men.

## Methods

### Ethical review and practices

This study was reviewed by the Science and Ethics committees of The Uganda Virus Research Institute and The University of Glasgow Faculty of Social Science and cleared by the Uganda National Council for Science and Technology. Informed consent was obtained from all interviewees and to enhance confidentiality pseudonyms are used for both the study area and participants.

### Study setting, sample and data collection

This paper is based on ethnographic research in Mam-Kiror and specifically on 26 interviews conducted with different categories of men. At the time of the study Mam-Kiror village had a population of about 750 people, predominantly Iteso by ethnicity and Catholics. The prevalence of HIV in Busia district was estimated to be 10%, which was by then above the national average of 6.4%, and anecdotal information from the district indicated that Mam-Kiror parish was one of the parishes with the highest HIV prevalence. While the majority of the women were engaged in subsistence farming, the main occupation of the men in the area at the time of research was artisanal gold mining. Gold mining as an occupation tends to shape men’s perceptions of masculinity in some distinct ways [27-29]. The health risks associated with this occupation, alongside the economic opportunities, cash payments and trading opportunities, worried the Busia district health authorities and in this study, these features provided scope to explore men’s way of life as it relates to their perception of masculinity, risk of HIV and their response to it.

The study sought to recruit a sample of men with roughly an equal proportion (7) from four sub-categories in relation to HIV, but due to some refusals, the eventual total sample size recruited included: nine men being treated for HIV, eight who tested but dropped out of treatment, seven not tested but who suspected HIV infection and three with health problems unrelated to HIV. The last group was included primarily to conceal the focus on men with HIV by undertaking the study with a more diverse group of men.

The 26 men interviewed were selected through purposeful and snowball sampling methods. Those receiving treatment and some who had dropped out of treatment, were accessed with the help of Busia Health Centre IV, a public treatment facility. Potential participants were first briefed about the study by their HIV counsellors and when they indicated willingness to learn more about the study, they were asked either to contact or be contacted by the researcher (GES), with the option of their counsellor being present, for a brief meeting to discuss their participation. Each potential participant was given sufficient time to ask questions and carefully consider whether they should be included in the study. Following a discussion of their rights, benefits and voluntary nature of their participation, 12 willing men were selected. The snowball process was facilitated by two of the participants receiving HIV treatment who knew others within the village who had either dropped out of treatment or who suspected that they were infected with HIV but had not tested.

The snowball approach is valuable in accessing a hidden population because the sampling emerges through a process of reference, whereby a participant refers the researcher to another potential participant who would otherwise not be known to the researcher [30]. Since accessing and interviewing men who had tested positive to HIV but had dropped out treatment or who suspected infection was very sensitive and the sample was hidden, the first introduction from referring participants enhanced the researchers’ credibility and acceptance with the potential participants, enabling us to recruit the required sample of men.

For every participant, written consent (signed or thumb print) was obtained following a detailed discussion of the study. However, despite assuring this standard procedure, not all men were keen to participate; some hesitated and required follow up, while others were less open to discuss their story initially. To increase the trust of the participant, GES sometimes explained to them that many other men in the village with similar characteristics were being interviewed, without naming those individuals, and suggested convenient places for the interviews to encourage them.

GES, who is from the same ethnic group but not from the same community, conducted both the in-depth interviews and participant observation, interacting with local people including the interviewees and collected data relating to social life generally and specifically on masculinity and health. The interviews were conducted either in men’s homes or other places of their choice, including work places, depending on whether the place assured adequate confidentiality. Interviews were based on a flexible topic guide, were audio recorded and transcribed. The interview guide included topics such as ‘what it means to be a man’, ‘HIV testing’ and ‘experiences of living with HIV/AIDS’. A shared language and culture positioned GES as a native interviewer enabling questioning on issues that were not spoken about regularly but were potent in people’s social relations.

### Data analysis

Thematic framework analysis [31] was performed and managed using Nvivo 8. The first author (GES) read the transcripts and field notes, identified preliminary themes and coded the data, following discussion with co-authors. Coded data were summarised in a matrix, comparing cases and identifying recurrent categories and similarities and differences. Pseudonyms are used to anonymise participants.

GES awareness of the local situation, because of his ethnicity, facilitated a reflective analysis of the data, but in addition, the other two authors (DW and JS) provided an external perspective, facilitating a deeper understanding of issues that might have been too familiar and taken for granted.

## Results

### Interviewee’s characteristics

The 26 interviewees were aged between 27 and 51 years old. Only three did not have children of their own at the time of the study. With the exception of two who had attained secondary education, all the other interviewees reported never completing primary school. All, except four, were not married at the time of the study, three of those being widowed or separated from their wives and one never married. Five of the married men had more than one wife. Although polygamists tended to command some attention and admiration, being most esteemed in male sub-cultures and social gatherings, poorer men or men known to be infected with HIV were more likely to be stigmatised for polygamy. They were considered to be unrealistic and taking on additional provider responsibilities which were beyond them. Ten of the men interviewed reported being actively involved in artisanal gold mining at the time of the interview but all the others had mined at some stage in their life. Most had multiple sources of livelihood but generally earned small incomes.

### Discourses about HIV testing: with whom do men discuss HIV testing?

Men evaluated their risk of HIV infection and talked about testing with various categories of people, including spouses, parents and friends, in different contexts. However, it was mostly outside the domestic sphere and with peers that men reviewed their sexual histories and jointly discussed their risk of HIV infection. Emmanuel (age 42, living with HIV) described why, before he sought a test, he first chose to talk about HIV risk with a friend:

[…] because he used to go there [for HIV treatment] and I said maybe I have the same problem like him. Also when you have a problem like this one, you tell a fellow man first because you know he can understand how it came about. You can talk about it with a friend and also talk about your movements [sexual contacts].

The expression of similar beliefs was observed at other times. In a conversation between two men (aged between 20 and 25) who were discussing whether to test during an HIV testing outreach event that was being conducted in their village in December 2009, one of them said to his friend: “Man, I really urge you; do not miss this chance as you know what [sexual contacts] we have had.”

In contrast, discussing sexual risk and HIV with a spouse or other family members was believed to be inappropriate and threatening to one’s masculine authority. While some men acknowledged that their wives were also inclined to extramarital relationships, it was generally believed that men were more promiscuous and therefore they tended to avoid discussing their sexuality with their wives, for fear of being blamed for bringing sickness. Compared to wives and other family members, friends frankly assessed and discussed their risk of being infected, since they were often privy to information about each other’s sexual relations. Sata (age 36, not tested but suspected HIV infection) narrated that whenever he shared his concerns about his health with friends, those friends usually pointed explicitly to his past risky sexual contacts:

Whenever I tell a friend that I am feeling pain all over the body, that friend just responds by saying, ‘why don’t you go for an HIV test?’ They [friends] can say ‘you have had many women and now you are pretending that you don’t know what is paining you!’ So it might be true [that I am infected] because when I look back, as a man… and yet friends are also saying that you used to bring women… and you never know you could get any disease!

Many of the accounts suggested that men tended to resent their wives and were quick to silence them if they attempted to discuss, or believed that their husbands might be at risk of HIV. This underscored the unequal power relations between partners as Solomon’s (age 42 years) story shows. When his wife suggested that he should go for an HIV test following a persistent cough, Solomon’s response was: “‘who told you that my sickness is AIDS?’ […] and she just kept quiet”. In contrast men’s reactions to their friends’ assessment of their HIV risk was generally positive, with minimal resentment, as the following remark by JB (age 37, not tested, suspects infection) highlights:

When it comes to those things (sexual affairs and risk), friends cannot be said to be accusing; they see everything but for a wife, she just guesses, so you can deny and ask her ‘have you seen me [with another woman]?’

Women’s accounts also confirmed that there were far fewer discussions about HIV risk and testing between them and their husbands. Discussions, more accurately quarrels, only increased if there were obvious symptoms or events which pointed to the possibility that both/one of them may be infected. The wife of Abraham (age 50, receiving treatment) responded as follows when we asked whether anybody in the family had talked to her husband about HIV testing before he undertook it: “Nobody, I think he himself [made the decision]; it must be from talking with his friends.” Like several others in the village, Abraham’s wife explained further that she did not suggest that her husband went for HIV testing because it was not their norm to discuss HIV as a couple.

### Negative perceptions of different testing options

HIV testing in Mam-Kiror was accessed through various options, including individual voluntary counselling and testing, couple testing, homes based HIV testing and community centre outreach testing. Each of these options was valued differently by men for various reasons. However, there was strong criticism of couple testing and community centre outreach testing. Men considered couple testing an unrealistic strategy that did not consider the common pattern of marital relationships and sexual lifestyles in the village. It was perceived as threatening to male authority and relations with their wives in three main ways. First, couple testing requires that both a man and a woman go together to a health provider for counselling and testing so that they know their HIV status together. However, many interviewees argued that with a tendency for men to pursue secret extramarital relationships, the requirement that testing is done as a couple would potentially lead to disclosure of those extramarital affairs. This risked severing relations with their partners or wives and undermining the masculine status associated with marriage. The anxiety about the potentially disempowering element of couple testing was also strongly expressed by other men in the village during casual conversations:

That [couple testing] is the most difficult thing. I keep hearing it over the radio that people to test with your friends [partners] but this is not easy. Many men are just cheating with other people’s wives so where will they test from. They do not want to be seen together, only meet at night (Man, age 20, February 2010).

Second, men were bothered about the necessity and practicalities of couple testing in steady marriages or in polygamous unions. As we saw, Solomon blatantly refused his wife’s suggestion that they go to test as a couple. Couple testing in polygamous marriages was believed to be unfeasible and needlessly demanding to the man, as one participant argued:

If you cannot test alone, can you now carry your wife to go and test? How many times will I go for testing if one has two or three women and then also others who are not his? Don’t you see that it will be difficult? (Man, aged 35, June 2010)

Also, as we report elsewhere [21] men argued that there was a tendency for some wives, who felt protected by the health worker during the counselling process, to show their power over their husbands, by questioning their misdeeds and blaming them for risking their lives with HIV. These men’s views about the challenges of couple testing did not appear to differ by age, with both older and younger men expressing similar worries about the possibility of a man being humiliated during couple testing.

Third, for other men the fear of undermining opportunities for sex was an important barrier to early testing with a non-marital partner. Several men, especially younger ones, said that in the context of widespread competition for sexual partners, insisting on a test together with a potential sexual partner meant that one lost the opportunity for sex, since other men did not demand a test.

Men were also very anxious about community centre outreach testing. While this option eliminated the barrier of distance and lack of knowledge of where to find testing services, the men we spoke to argued that community centre outreach testing was potentially problematic to their social relations, hence making men reluctant to test. This approach introduced a distinctively undesirable obstacle, the public monitoring of testing in the village. Some villagers watched to see who tested and who did not and why. In some of their accounts, men said that both deciding and declining to get tested prompted speculation among fellow villagers, who evaluated those individuals’ sexual contacts, sometimes making damaging allegations about them. In many ways this informal monitoring of testers and non-testers was stigmatising and men were often the prime targets of this scrutiny because, generally, they were more likely to have had extramarital relationships and were, therefore, believed to need testing, as illustrated by the following extract from a conversation:

There are some people in the village who I know are sick but have never tested, so the other time when the testing service was brought to the Centre, I went there to see if they would come for testing but I did not see them. Many of them fear and these are mostly men, they fear a lot because they know their behaviour (Man, age 50, March 2010).

While a few men said that they would not mind the public learning that they undertook testing during a community testing event, they appeared to assess their own risk of infection as low. A 25 year old man who gave the impression that his sexual behaviour had not put him at risk discussed this as follows:

Now what will I fear? I cannot fear anything [regarding HIV testing in public]. Those who fear, know that their movement [sexual network] is bad. You see there are some men who you can just know that they have a problem (Man, age 25, December 2009).

### Mismatch between scheduling of testing and work schedules

The organisation and scheduling of testing services, particularly in the public health centres, was a major obstacle to testing among men. In most public facilities HIV testing and counselling has been fixed to specific days of the week and times of the day which potential users have to know. However, because details about these services were not usually advertised at the community level, some men reported being turned away when they went on the wrong day/time, being told: “Sorry you are late, come back tomorrow,” or “we do not test people on this day, come back another day,” when they visited facilities to get tested. Given that many men were very concerned about repeatedly missing their work, this resulted in missed opportunities for testing as Elly (age 27), one of the men who had contemplated testing, put it: “Also work, I don’t have time; I have to look for what to feed the family with.” When observing testing in a public facility, we witnessed two men (approximately in their 20’s) who arrived late (in the afternoon) for testing being turned away by a counsellor who explained that the lab and counselling services were already closed. She advised them to come back early the same day of the next week.

Men also decried the absence of safe parking lots for their bicycles or motorcycles in many of the health facilities; describing these facilities as “inhospitable” or “unwelcoming” and citing it as a hindrance to making facility visits. Many said that they were expected to park their bicycles under an unguarded tree within or outside the facility, risking theft. This was the unfortunate experience of David (not interviewed), who borrowed a bicycle to pick up his HIV medicines but the bicycle got stolen at one of the public health facilities in the nearest town. Despite his explanations and plea for forgiveness, he was made to pay the owner for the bicycle.

### The tendency to associate HIV symptoms with the impact of work

Men tended to associate early symptoms of HIV, especially prolonged cough and body aches, with the heavy work they did and therefore saw no need to immediately seek a test. Solomon narrated that he had put off seeking a test, despite acknowledging that his sexual behaviour could have exposed him to the risk of HIV, suffering with a chronic cough and being advised by friends to seek an HIV test:

It [chest and cough] just started to pain […] I think it may be the heavy work which I did while still physically strong. […] you could go to dig deep underground to mine gold and then also the smoke from the kerosene candles used inside there [tunnels], I think it is what brought this pain in the chest.

The men not only associated their symptoms with their hazardous work, but also easily explained them away, especially the persistent coughs, as “everybody’s illness”, since it was rampant among miners. Mike (age 31, receiving HIV treatment) was an interesting example. He described his experience with symptoms as follows:

I didn’t know which type of disease I was suffering from! For me I thought maybe it’s because of the heavy work I was doing, of crushing stones [gold ore] or that it is the dust which has affected me, so I did not do much [to check] because I was seeing it as a common problem here.

### Resilience and unwillingness to admit to problems

Men were reluctant to acknowledge symptoms and consider them serious enough to warrant seeking prompt care. This attitude was attributed to the social construction of a man as independent and resilient. Most narratives from both the general public and the men interviewed suggested that men generally take longer than women to admit failure to resolve a health problem, even when it is obviously overwhelming them, because socially it is important for a man to be self-reliant. Men’s reluctance to admit problems was explained by the desire to show that they are in control of the situation. Those who believed that they still looked physically strong and had not been incapacitated saw no need to immediately seek testing. This perception is illustrated in Solomon’s evaluation of his health and decision to delay help seeking: “[…] the energy is there except the pains I am experiencing; but [for] AIDS, I hear it completely weakens one.”

It was widely asserted that not testing was an intrinsic expression of masculine courage and self-control amidst a crisis. Due to this attitude, some men considered their delay to start HIV treatment as an achievement that attested to their enduring physical strength and courage despite the symptoms. However, this sense of achievement and esteem resulting from delayed testing varied by occupation and by age. For example, the stories of Juma and Emma (age 42) suggest that men whose current employment was artisanal gold mining were more anxious about the low esteem resulting from failure to demonstrate resilience and the courage of a man against testing and admitting to being ill, than men from other local occupations. This was perhaps because their occupation relied heavily on collaborative work where physical strength and a hard work ethic were encouraged and highly valued. For instance, both Solomon and Juma delayed testing because they still had bodily strength and both also repeatedly discussed how they were known to be very strong men at work. Overall, however, the younger men were more anxious about failure to demonstrate independence through strength than the older men. As they grew older, men tended to have a more flexible and resigned attitude towards strength/resilience as a means to express themselves, arguing that they were judged by other parameters such as an ability to support their family and their previous achievements. Some older men felt more assured about coping with HIV diagnosis because of the resources they had. Solomon’s story highlights this. He attempted to disassociate himself from the need to demonstrate his masculine resistance to testing by arguing that if he tested and people attempted to discriminate against him, he would not be bothered by it because:

I have had most things on this earth whether it is money […], whether it is land, […] there is some money I have saved in the form of a plot (of land) which I had bought for children. So I can decide to sell part of it, if things are bad.

### How do notions of masculinity prompt testing among men?

While many factors delayed or prevented men from testing all together, there were other factors and circumstances linked to their masculinity that created an urgent need for men to test.

### The need to access HIV treatment

Many men perceived the main purpose of testing as a way to access treatment. In many of the informal conversations, most people believed that HIV prevalence was very high in their village, especially among men. Men often stated: “We are all sick here; you think people here are healthy! There are just few people who are okay.” They argued that men in this area had a high risk of being infected since they tended to have many extramarital relationships. In part this belief that many of them were already infected contributed to a reluctance to test. One man (aged 36) described thus: “If the thing [HIV] is already there [in the body] it will just come out by itself [so there’s no need to test].” However, several interviewees, as well as men from the general population, repeatedly stated that they would consider testing only if they have come to the final conclusion that they had HIV and had reached the stage of needing treatment:

For me I think I can only test after I have fallen sick a number of times and I know that I am now infected with slim, so that I can start taking the medicine (Man, age 32, December 2009).

### Family responsibilities

Men’s accounts about the need for testing were linked to the need to secure their masculinity as family carers. Men understood that HIV treatment extended life, thereby enabling them to continue fulfilling their masculine roles, although some did not refer explicitly to fatherhood obligations as the major factor motivating them to test. While some men who assumed they were already infected saw no immediate need for an HIV test, many did not wait until symptoms were obvious and the illness was serious, indicating the need to test in order to access treatment. Responsibilities and obligations for children were the main reasons for wanting to “test and live a few more years” as Tony (age 39, who had not tested but suspects HIV infection) described. He argued that he has to get organised and start treatment to give him some additional years to raise his children. Furthermore, his strong sense of obligations to other kin or people who had helped in the course of the illness provided the motivation to agree to an HIV testing. Jeremiah’s (age 40, receiving treatment) experience was another striking example of this. Prior to him getting tested, his brothers offered different kinds of material and medical help: purchasing medicines for treatment of symptoms, a trip to the city for better care at a brother’s home, another brother accompanying him to hospital to test/initiate HIV treatment and another lending his motorcycle for the transportation. Jeremiah and other men like him, felt that a demonstration of a sense of responsibility towards their supportive relatives had to be shown by agreeing to get tested.

### Debilitating illness

In recounting their experiences, many of those who tested or contemplated testing, described illnesses that significantly impaired their ability to work and/or to socialise with others in the public arenas, which created an urgent need to test. Among the many interesting examples was the account of one man (in his 30’s) during a conversation about this topic in December 2009: “For me I think I can test after I have fallen sick and I know that I am now infected with slim [AIDS], so that I can start taking the medicine.”

In Solomon’s story, we saw that he was not ready to test because he still had energy, in spite of the cough persisting, suggesting that having less debilitating illness delays readiness to test. However, in a subsequent conversation his narrative appeared to confirm the role of an illness in influencing his desire to test saying:

The illness has kept me coiled-up in bed and at home for weeks; I cannot work and [cannot] even go out to the centre to be with others. So I think where it has reached, I need to check.

### Death of spouse or rival sexual partner

Many accounts revealed that the death of spouse or of a rival sexual partner increased the likelihood that a man might have contracted HIV and therefore needed to check. Ben was a good example of those testing following the death of a spouse. His own symptoms did not prompt testing but the death of his wife did:

It was in 2008 when I lost her [wife]. She was very sick, only for one month and unfortunately passed away. After burying her, I got concerned about my health because before that I was not feeling well. I was feeling a lot of unexplained pain all over the body even before she died and so I decided to go for testing.

For men such as Tony (age 39) and JB (age 37) (both contemplating testing) and Mike (already tested), the death from AIDS or serious sickness of men who had had a sexual relationship with the same woman as them was the reason for their determination to get tested immediately. JB explained:

Although I have been feeling sickly and a bit concerned that I might have a problem, the death of Mr Micah last week due to this disease [AIDS] has made me more worried because some time back, he also used to go to the same woman I used to go to. So, I also now want to go and check this body.

### Pressure from male friends

Many of the stories we heard about men’s experiences of, or intentions about, HIV testing underlined the role of friends/workmates, although there were some counter-examples such as Abraham (age 50) who got tested through personal determination rather than involvement of other parties. As illustrated in many individual and group accounts in our data, peers with whom one worked or interacted regularly easily recognised symptoms and recommended testing and sometimes offered to “escort” them to test. Tony described coming under pressure from work friends: “[…] they [friends] are always telling me to first test, because they see that I have no energy, I no longer go to dig with them as a group etc.” Paul, another man who had not yet tested, also provided an insightful comment that distinctively illustrates the strong beliefs and respect some men have of their peers, saying; “I would have gone if a friend took me.” In other accounts, it was evident that there were men who believed that knowing friends/colleagues who had tested or wished to test would motivate them to get tested too. They argued that it is less stigmatising to test together, “especially if you are good friends, with a similar sexual history”. For example, during a conversation between three men who were discussing the impact of knowing that a friend had tested, one of them said:

“You know for us men, you first want to hear that your friend also went [for HIV testing] or has started [taking ART] medicine then you can say, aaah, let me also go.”

Men who had publicly disclosed and were receiving treatment were especially instrumental in encouraging others to get tested. For example, Noah, Abraham and Isaac said that they not only had encouraged men to test but frequently received requests from friends for advice. Isaac (age 37) narrated:

There are men who come to me asking me ‘man, which hospital did you go to?’ For example someone may be having boils etc., they fear to discuss with their wives, so they come to a friend like me. So I can maybe tell them that today is not a day for testing but you get ready I will take you there.

Nonetheless, in all these discourses about the role of friends, trust was important because men were concerned about disclosure. The participants who had greater trust in their colleagues and were not worried about being discriminated against by them, were less reluctant to test and disclose. These were often the older men, such as Noah, age 50, Silver, age 50, Salim, age 45 and Emma age 42.

## Discussion

This paper discusses the factors that influence men’s HIV testing in a rural area and how they are intertwined with themes of masculinity. Our previous analysis of masculinity suggested two main categories of masculinity in Mam-Kiror village, one based on reputation and the other on respectability, although each had various ideals that overlapped [25]. The ideals of masculinity linked to reputation included sexual achievement and fathering many children, physical strength and resilience, a work ethic, independence and power, socialising with others and a requirement to spend on leisure. Respectable masculinity was comprised of ideals such as marriage, fathering children and providing for them, sexual fidelity, demonstration of wisdom and respect of self and others. We argue here that the different forms of masculine esteem lead to different motivations towards HIV testing.

Our findings suggest that the current norms, practices and contexts for testing, particularly couple testing and community outreach testing, largely tend to challenge and undermine the prevailing values of masculinity in the study setting, undermining men’s readiness to seek HIV testing. For instance, besides being perceived as needlessly demanding and impractical, especially in polygamous marriages, couple testing was threatening to men’s masculinity because it meant disclosing their extramarital relationships. Due to the fears around upsetting stable marital and family relations, men were especially unwilling to discuss HIV with their wives. Several studies in SSA have described men’s tendency to avoid HIV services for fear of their wives’ negative reactions if they were found to have had extra-marital relationships and risked bringing HIV to the family [17,32,33]. As Skovdal et al. [33] argue, such fear leads men to deny their risky sexual behaviour and refute the presence of HIV, undermining their uptake of HIV testing. Men tend especially to dislike couple testing if their wives mistrust them since it has the potential to further destabilise marriages, as found in a study on couple testing in antenatal care in east central Uganda [34]. Given that marriage was an important masculine ideal shared by both men amongst themselves and the wider society in the study setting, divorce or separation from one’s wife greatly undermined a man’s public and domestic masculinity. The overwhelming concern about the disempowering nature of couple testing and the negative impacts of a positive test on their relations with wives did not seem to differ by age: both younger and older men in the study area expressed this fear. We also found that men, especially older ones (aged about 40+), feared that if they tested HIV positive, their relatives, particularly their wife’s relatives, would question their morals, undermining their respectability.

An unexpected finding in this study was the view by some men that couple testing undermined their opportunities for sex, hindering testing with a non-marital partner before sex. Subscribing to the masculinity of sexual achievement meant that men in this area competed intensely for and frequently shared, sexual partners. It was especially important for one’s masculinity to be the first to have a sexual relationship with a new woman in the village because this often implied superior seduction skills and/or use of money. Thus, several men argued that insisting on an HIV test with a partner in the context of widespread competition for sexual partners not only affected one’s chances of having sex since other men did not demand it, but also greatly undermined the reputation attained from others as a powerful rival sexual partner. In addition, the sense of superiority achieved from a successful predatory sexual relationship with another man’s partner also complicated the requirement for early testing among men.

Men’s anxiety regarding participating in the community centre outreach testing was also articulated in relation to their sexuality and risk of being negatively labelled by the community, which damaged their social relations and established position in the society. Organisational and infrastructure arrangements that do not ensure privacy during HIV testing have been criticised in many settings, including Uganda, for discouraging potential testers. A common concern is that visitors to anywhere identified as a testing facility are thought to be worried about HIV, leading to rumours and stigmatisation [35,36]. While the fears about public monitoring of testers and non-testers and inadvertent disclosure during a community HIV testing event may not be unique to men, in a setting such as Mam-Kiror where many people know each other and men are believed to be likely to have more sexual partners than women, their decision whether or not to test may attract greater public interest. Although some men in our study argued that they would not mind the public learning that they undertook testing during a community testing event, these particular men assessed their own risk of infection as low. This suggests that some men may have confidence to test publicly to prove their sero-negativity but might want to avoid testing in the presence of others if they expect a positive HIV test result. However, in some cases, what might distinguish such people is usually their decision to ignore what others think [36].

We found that not admitting to problems or postponing them until one was severely affected was an important measure of one’s resilience and masculine strength. This supports the argument that the health related decisions of men (and women) is a means of constructing their gender identities as frequently discussed in various literature [22,37,38]. However, for men, many of the stages involved in seeking professional health care, such as admitting the need for help and recognising a problem, tend to conflict with the messages that they receive about the importance of self-reliance, emotional control and physical toughness [39]. Therefore, HIV test seeking behaviour may also be understood as an expression of weakness where male strength is expected [40]. This might also illuminate men’s tendency to explain away symptoms as common hazards of their work, despite some suspecting it was HIV. The inflexible testing schedules which threatened men’s work, not only affected earnings but also undermined one’s reputation as a hard worker, leading some men to fail to honour their appointments.

There were factors that interacted with respectable masculinity and to some extent reputational masculinity to encourage testing either directly or indirectly. Symptoms that threatened work, the death of, or serious illness of, a rival sexual partner or spouse and pressure from peers who observed symptoms, were important. Although seeking a test for these reasons might be of little value for prevention of infection by the man, since infection might have already occurred by then, such testing is useful for treatment initiation. Some men therefore saw no point in testing unless one was intending to get treatment. Men understood that treatment extended life and so it offered a second chance for one to continue performing his masculine roles. The respectable masculine ideal to fulfil responsibilities and obligations to family was a strong motivator to seeking an HIV test by men.

We found that the influence of peers in getting tested was important to men. Collegial discussion of the risk of HIV infection and the need for medical treatment were common and appeared to be highly valued as those people often knew one’s sexual networks. It was less stigmatising and encouraging to test together with a good friend with a similar sexual history, although they were concerned about disclosure.

Overall, men’s narratives positioned HIV testing as a social process understood within the social context and relationships men engaged in, rather than an entirely self-determined enterprise. Their decision as to whether to test for HIV was influenced and negotiated in complex ways with many stake holders. These findings suggest that there is need for HIV policy makers and support agencies to review how various testing options might marginalise men from seeking testing services and address the barriers that hinder access.

We recognise that generalizability is limited by the small sample size and highly contextual nature of the participants’ accounts and setting. Our line of interview and interaction with the men could have also created an improved sense of the importance of health and may have led some men to portray themselves differently, in order to construct what they perceived would be a more socially desirable image. However, participant observation data were collected to triangulate with the data from the interviews. The strength of our study is that the ethnographic focus on men privileges their often nuanced and muted voices with regard to HIV testing and it raises important questions that may be asked in many other settings.

## Conclusion

In the rural setting where this study was conducted, characterised by collective life styles, social ties and decision making, men’s decisions to test for HIV should be understood as a social process influenced by different masculine ideals. We found that the two main forms of masculine ideals prevailing in Mam-Kiror in Busia led men to have different motivations towards HIV testing. Reputational masculinity was largely inconsistent with the requirements of couple testing, community outreach testing and the organisation of testing services, discouraging men’s testing. Conversely, concern to perform one’s family roles as a respectable man meant accessing treatment to extend one’s life, which encouraged men to test. HIV policy makers and support agencies should reflect on how various testing options might marginalise men from seeking testing services and address the barriers that hinder access.

## Competing interests

The authors declare that they have no competing interests.

## Authors’ contributions

GES conceptualised the study, collected the data, conducted analysis and drafted the manuscript. DW and JAS advised on design and analysis and reviewed the draft manuscript. All the authors read and approved the final manuscript.

## Authors’ information

Godfrey E. Siu, PhD, Lectures in Child Health and Development Centre, College of Health Sciences, Makerere University, Uganda and is a Post-Doctoral Social Scientist, MRC/Uganda Virus Research Institute, Uganda. His research focuses on gender, masculinity, young people and HIV. Recent publications include: Dividuality, masculine respectability and reputation: How masculinity affects men’s uptake of HIV treatment in rural eastern Uganda (*in Social Science & Medicine 2013*). How a masculine work ethic and economic circumstances affect uptake of HIV treatment: experiences of men from an artisanal gold mining community in rural eastern Uganda (*in J. International AIDS Society 2012*). HIV serostatus disclosure and lived experiences of adolescents at the Transition Clinic of the Infectious Diseases Clinic in Kampala, Uganda: a qualitative study (*in AIDS Care 2012*).

Daniel Wight is Professor and leads the children, Young people, Families and Health Programme in the MRC Social and Public Health Sciences Unit, Glasgow, UK. Having specialised in young people’s sexual health, current research include: parent–child relationships, gender, developing research capacity in Africa and the development of evaluation of behavioural interventions. Recent publications include: Young people’s lives and sexual relationships in Rural Africa (*Lexington Books 2012*); The need to promote behaviour change at the cultural level, *(in BMC Public Health 2012*) and A Review of Interventions with Parents to promote the Sexual Health of their Children *(in Journal of Adolescent Health 2013*).

Janet Seeley is Professor of International Development, School of International Development, University of East Anglia, Norwich, UK and Head of Social Science Programme, MRC/UVRI Uganda Research Unit on AIDS. Her research focuses on the social aspects of HIV, poverty and gender and she has published widely on these topics. Among her recent publications are `High HIV Incidence and Socio-Behavioral Risk Patterns in Fishing Communities on the Shores of Lake Victoria, Uganda (*in Sexually Transmitted Diseases 2012*) and How HIV diagnosis and disclosure affect sexual behavior and relationships in Ugandan fishing communities (*in Qualitative Health Research 2013*).

## Pre-publication history

The pre-publication history for this paper can be accessed here:

http://www.biomedcentral.com/1471-2458/14/33/prepub

## References

[B1] WHO, UNAIDS, and UNICEF Towards Universal Access. Scaling Up Priority HIV/AIDS Interventions in the Health Sector: Progress Report 2007 Geneva: WHO Press

[B2] Ministry of Health (MoH) [Uganda] Uganda AIDS Indicator Survey 2011 2012 Kampala

[B3] Njeru M Practicing provider-initiated HIV testing in high prevalence settings: consent concerns and missed preventive opportunities BMC Health Serv Res 2011 11 1 87 10.1186/1472-6963-11-87 21507273 PMC3105945

[B4] Msuya SE Low male partner participation in antenatal HIV counselling and testing in northern Tanzania: implications for preventive programs AIDS Care 2008 20 6 700 709 10.1080/09540120701687059 18576172

[B5] Peltzer K Determinants of knowledge of HIV status in South Africa: results from a population-based HIV survey BMC Public Health 2009 9 174 1 11 19500373 10.1186/1471-2458-9-174 PMC2700104

[B6] Mitchell S Equity in HIV testing: evidence from a cross-sectional study in ten Southern African countries BMC Int Health Hum Rights 2010 10 1 23 10.1186/1472-698X-10-23 20836859 PMC2945979

[B7] Mwakiza I Who has access to counselling and testing and anti-retroviral therapy in Malawi - an equity analysis Int J Equity Health 2009 8 13 1 9 19416512 10.1186/1475-9276-8-13 PMC2683850

[B8] Braitstein P Gender and the use of antiretroviral treatment in resource-constrained settings: findings from a multicentre collaboration J Womens Health (Larchmt) 2008 17 47 55 10.1089/jwh.2007.0353 18240981

[B9] Hult JR Maurer SA Moskowitz JT “I'm sorry, you’re positive”: a qualitative study of individual experiences of testing positive for HIV AIDS Care 2009 21 2 185 188 10.1080/09540120802017602 19229687

[B10] Matovu JKB Makumbi FE Expanding access to voluntary HIV counselling and testing in sub-Saharan Africa: alternative approaches for improving uptake, 2001–2007 Trop Med Int Health 2007 12 11 1315 1322 10.1111/j.1365-3156.2007.01923.x 17949401

[B11] Hutchinson PL Mahlalela X Utilization of voluntary counseling and testing services in the Eastern Cape,South Africa AIDS Care 2006 18 5 446 455 10.1080/09540120500213511 16777636

[B12] Weiser SD Routine HIV testing in Botswana: a population-based study on attitudes, practices, and human rights concerns PLoS Med 2006 3 7 e261 10.1371/journal.pmed.0030261 16834458 PMC1502152

[B13] Ostermann J Who tests, who doesn’t, and why? Uptake of mobile HIV counseling and testing in the Kilimanjaro region of Tanzania PLoS One 2011 6 1 e16488 10.1371/journal.pone.0016488 21304973 PMC3031571

[B14] Mugisha E van Rensburg GH Potgieter E Factors influencing utilization of voluntary counseling and testing service in kasenyi fishing community in Uganda J Assoc Nurses AIDS Care 2010 21 6 503 511 10.1016/j.jana.2010.02.005 20381382

[B15] Bila B Egrot M Gender asymmetry in healthcare-facility attendance of people living with HIV/AIDS in Burkina Faso Soc Sci Med 2009 60 14 854 861 19539415 10.1016/j.socscimed.2009.05.035

[B16] Obermeyer CM Gender and HIV testing in Burkina Faso: an exploratory study Soc Sci Med 2009 69 6 877 884 10.1016/j.socscimed.2009.07.003 19631435 PMC4260152

[B17] Bwambale FM Ssali SN Byaruhanga S Voluntary HIV counselling and testing among men in rural western Uganda: implications for HIV prevention BMC Public Health 2008 8 263 1 12 18664301 10.1186/1471-2458-8-263 PMC2529297

[B18] Gage AJ Ali D Factors associated with self-reported HIV testing among men in Uganda AIDS Care 2005 17 2 153 165 10.1080/09540120512331325635 15763711

[B19] Obermeyer CM Osborn M The utilization of testing and counseling for HIV: a review of the social and behavioral evidence Am J Public Health 2007 97 10 1762 1774 10.2105/AJPH.2006.096263 17761565 PMC1994175

[B20] Peacock D Men, HIV/AIDS, and human rights J Acquir Immune Defic Syndr 2009 51 S119 S125 10.1097/QAI.0b013e3181aafd8a 19553779 PMC2853958

[B21] Kimmel MS Kimmel MS Rethinking “masculinity”: new directions in research Changing Men: New Directions in Research on Men and Masculinity.Sage Focus Editions, Vol 1987 Thousand Oaks, CA, US: Sage Publications, Inc 9 24

[B22] Courtenay WH Constructions of masculinity and their influence on men’s well-being: a theory of gender and health Soc Sci Med 2000 50 10 1385 1401 10.1016/S0277-9536(99)00390-1 10741575

[B23] Connell R Gender, health and theory: conceptualizing the issue, in local and world perspective Soc Sci Med 2011 2011 1 9 10.1016/j.socscimed.2011.06.006 21764489

[B24] Flood M Pathways to manhood: the social and sexual ordering of young men’s lives Health Educ Austr 2002 2 24 30

[B25] Siu GE Seeley J Wight D Dividuality, masculine respectability and reputation: How masculinity affects men’s uptake of HIV treatment in rural eastern Uganda Soc Sci Med 2013 89 45 52 23726215 10.1016/j.socscimed.2013.04.025

[B26] Wilson PJ Reputation and respectability: a suggestion for Caribbean ethnology Man 1969 4 1 70 84 10.2307/2799265

[B27] Cartier LE Bürge M Agriculture and artisanal gold mining in Sierra Leone: alternatives or complements? J Int Dev 2011 23 8 1080 1099 10.1002/jid.1833

[B28] Perks R ‘Can I go?’—exiting the artisanal mining sector in the democratic republic of Congo J Int Dev 2011 23 8 1115 1127 10.1002/jid.1835

[B29] Siu GE Wight D Seeley J How a masculine work ethic and economic circumstances affect uptake of HIV treatment: experiences of men from an artisanal gold mining community in rural Eastern Uganda J Int AIDS Soc 2012 Supplement 1 15:17368 1 9 22713356 10.7448/IAS.15.3.17368 PMC3499901

[B30] Denscombe M The Good Research Guide for Small-Scale Research Projects 2010 MacGrawHill: Open University Press

[B31] Ritchie J Lewis J Qualitative Research Practice: A Guide for Social Science Students and Researchers 2003 London: SAGE Publications

[B32] Wyrod R Masculinity and the persistence of AIDS stigma Cult Health Sex 2011 13 4 443 456 10.1080/13691058.2010.542565 21246426 PMC3053429

[B33] Skovdal M Masculinity as a barrier to men’s use of HIV services in Zimbabwe Globalization Health 2011 7 1 13 10.1186/1744-8603-7-13 21575149 PMC3107786

[B34] Larsson E Mistrust in marriage-reasons why men do not accept couple HIV testing during antenatal care- a qualitative study in eastern Uganda BMC Public Health 2010 10 1 769 10.1186/1471-2458-10-769 21167040 PMC3018443

[B35] Jurgensen M The burden of knowing: balancing benefits and barriers in HIV testing decisions. a qualitative study from Zambia BMC Health Serv Res 2012 12 2 1 11 22222028 10.1186/1472-6963-12-2 PMC3268706

[B36] Wolff B Evaluation of a home-based voluntary counselling and testing intervention in rural Uganda Health Policy Plan 2005 20 2 109 116 10.1093/heapol/czi013 15746219

[B37] Galdas PM Cheater F Marshall P Men and health help-seeking behaviour: literature review J Adv Nurs 2005 49 6 616 623 10.1111/j.1365-2648.2004.03331.x 15737222

[B38] Verdonk P Seesing H de Rijk A Doing masculinity, not doing health? a qualitative study among dutch male employees about health beliefs and workplace physical activity BMC Public Health 2010 10 712 1 14 21092090 10.1186/1471-2458-10-712 PMC2998494

[B39] Addis ME Mahalik JR Men, masculinity, and the context of help seeking Am Psychol 2003 58 1 5 14 12674814 10.1037/0003-066x.58.1.5

[B40] Greig A Gender and AIDS: time to act AIDS 2008 22 S35 S43 doi:10.1097/01.aids.0000327435.28538.18 18641466 10.1097/01.aids.0000327435.28538.18 PMC3356155

